# Role of Prealbumin in Predicting the Prognosis of Severely and Critically Ill COVID-19 Patients

**DOI:** 10.4269/ajtmh.21-0234

**Published:** 2021-08-09

**Authors:** Ningning Cui, Haihui Tong, Yan Li, Yanyan Ge, Yuxin Shi, Ping Lv, Xiaobo Zhao, Jianchu Zhang, Gui Fu, Yanfen Zhou, Ke Jiang, Nengxing Lin, Tao Bai, Runming Jin, Sheng Wei, Xuefeng Yang, Xin Li

**Affiliations:** 1 Department of Nutrition and Food Hygiene, Hubei Key Laboratory of Food Nutrition and Safety, MOE Key Laboratory of Environment and Health, School of Public Health, Tongji Medical College, Huazhong University of Science and Technology, Wuhan, Hubei, P.R. China;; 2 Department of Paediatrics, Union Hospital, Tongji Medical College, Huazhong University of Science and Technology, Wuhan, Hubei Province, P.R. China;; 3 Department of Respiratory and Critical Care Medicine, Union Hospital, Tongji Medical College, Huazhong University of Science and Technology, Wuhan, Hubei Province, P.R. China;; 4 Department of Thoracic Surgery, Union Hospital, Tongji Medical College, Huazhong University of Science and Technology, Wuhan, Hubei Province, P.R. China;; 5 Department of Dermatology, Union Hospital, Tongji Medical College, Huazhong University of Science and Technology, Wuhan, Hubei Province, P.R. China;; 6 Department of Gastroenterology, Union Hospital, Tongji Medical College, Huazhong University of Science and Technology, Wuhan, Hubei Province, P.R. China;; 7 Department of Epidemiology and Biostatistics, Ministry of Education Key Laboratory of Environment and Health, School of Public Health, Tongji Medical College, Huazhong University of Science and Technology, Wuhan, Hubei, China

## Abstract

Most critically ill patients experience malnutrition, resulting in a poor prognosis. This study aimed to evaluate the association of prealbumin (PAB) with the prognosis for severely and critically ill coronavirus disease 2019 (COVID-19) patients and explore factors related to this association. Patients with laboratory-confirmed COVID-19 from West Campus of Union Hospital in Wuhan from January 29, 2020 to March 31, 2020 were enrolled in this study. Patients were classified into the PAB1 (150–400 mg/L; *N* = 183) and PAB2 (< 150 mg/L; *N* = 225) groups. Data collection was performed using the hospital’s electronic medical records system. The predictive value of PAB was evaluated by measuring the area under the receiver-operating characteristic (AUROC) curve. Patients were defined as severely or critically ill based on the *Guidance for COVID-19* (7th edition) by the National Health Commission of China. During this analysis, 316 patients had severe cases and 65 had critical cases. A reduced PAB level was associated with a higher risk of mortality and a longer hospital stay. The AUROC curve for the prognosis based on the PAB level was 0.93, with sensitivity of 97.2% and specificity of 77.6%. For severe cases, a lower level of PAB was associated with a higher risk of malnutrition, higher NK cell counts, and lower B lymphocyte counts; these factors were not significant in critical cases. C-reactive protein and nutritional status mediated the association between PAB and prognosis. This retrospective analysis suggests that the PAB level on admission is an indicator of the prognosis for COVID-19.

## INTRODUCTION

During early December 2019, several cases of pneumonia of unknown etiology were reported in Wuhan, Hubei Province, China.^1^^,^^2^ Severe acute respiratory syndrome coronavirus 2 (SARS-CoV-2) was identified as the pathogen of coronavirus disease 2019 (COVID-19).^2^^,^^3^ COVID-19 is a highly infectious and contagious disease that quickly spread to more than 55 countries worldwide within 3 months.^4^ As of January 24, 2021, the cumulative number of reported cases was more than 98.2 million and there were more than 2.1 million deaths globally since the start of the pandemic.^5^ Moreover, approximately 15% to 20% of COVID-19 cases progressed to severe cases.^6^ Notably, severely and critically ill patients usually developed acute respiratory distress syndrome (ARDS) or organ failure,^7^ thus leading to higher mortality.^6^^,^^8^ During an early study, approximately 17% of patients developed ARDS and 11% of patients had worsened conditions during a short period of time and died of multiple organ failure.^2^

The outbreak was declared a public health emergency of international concern (PHEIC),^9^ thereby placing health authorities on high alert worldwide. Although 1 year has passed, the global pandemic is still not under control, with new deaths occurring every day. Therefore, it is valuable to manage COVID-19 patients selectively according to the predicted prognosis using a sensitive and convenient indicator during an early stage. Serum prealbumin (PAB) presented a significant change during the early stage of SARS-CoV-2 infection.^10^ Previous studies have shown that PAB has been used to predict the disease severity and prognosis of COVID-19 patients.^10^^–^^13^ Additionally, the short biological half-life of PAB has a high degree of reactivity to the protein status.^14^ Therefore, PAB has been considered a good indicator of nutritional status that involves uncomplicated measurements.^15^ Malnutrition is common for critically ill patients^16^ and is related to increased mortality and length of hospital stay.^17^ A higher risk of mortality was also observed for COVID-19 patients with a higher nutritional risk screening 2002(NRS) score.^18^ Moreover, PAB was defined as an indicator of malnutrition, inflammation, and impaired immunity with other diseases.^15^ PAB might be particularly attractive for early risk stratification because it is a biomarker for malnutrition, inflammation, and immune state. Because of the influence of cytokines and immune status on the prognosis for COVID-19 patients,^19^^,^^20^ we hypothesized that cytokines and immune status may have a mediation effect on PAB and the prognosis.

PAB has been used as a valuable and effective indicator for predicting the prognosis and nutrition therapy for critically ill patients.^21^^–^^23^ However, its predictive power for COVID-19 patients has been inconsistent.^13^^,^^23^ A clear understanding of the role PAB in the progression of COVID-19 would help physicians to identify patients with a poor prognosis during an early stage of disease so they could be transferred to tertiary centers, which may facilitate the smooth progress of clinical practice and improve the prognosis of the patients. This study was conducted to evaluate the predictive ability of PAB for the COVID-19 prognosis and comprehensively analyze the association of PAB with the malnutrition risk, inflammatory markers, and immune status.

## MATERIALS AND METHODS

### Study design and participants.

This was a single-center, retrospective, observational study involving patients with COVID-19 cases confirmed between January 29, 2020 and February 19, 2020 (Supplemental Figure 1). Data were extracted from the West Campus of Union Hospital, Tongji Medical College, Huazhong University of Science and Technology (Wuhan, China), which is a designated hospital for COVID-19. A total of 408 eligible subjects were included for the analysis. These inpatients were diagnosed using a nucleic acid–positive test and classified as severely ill or critically ill. According to the protocols promulgated by the National Health Commission of China,^24^ severely ill patients were classified according to the following criteria: respiratory distress (respiratory rate ≥ 30 breaths/min); pulse oxygen saturation ≤ 93% on room air; and low arterial oxygenation ratio (PaO_2_/fraction of inspired oxygen ≤ 300). Critically ill patients were defined using the following criteria: respiratory failure requiring a form of mechanical ventilation; shock; and complications associated with other organ failure that require monitoring and treatment in the intensive care unit (ICU).

This trial was registered in the Chinese Clinical Trial Registry (ChiCTR2000030803) and approved by the Ethics Committee of Union Hospital Affiliated to Tongji Medical College, Huazhong University of Science and Technology ([2020]0096-1). Informed consent was waived because of the emerging infectious disease and retrospective study design. This study was performed in accordance with the Declaration of Helsinki.

### Data collection.

Anonymous clinical data were extracted from electronic medical records. At baseline, basic information (age, sex, and comorbidities), medical history (onset date, symptoms from onset to admission, chest computed tomography examination results, nucleic acid test results), and laboratory test results (routine blood and biochemical tests) were collected. All these data were obtained within 7 days of admission. Furthermore, data regarding the clinical outcome and hospital length of stay were collected until March 31, 2020, which was the last follow-up date.

### Laboratory tests.

All laboratory tests were performed using standard clinical chemistry methods.^18^ A BC-6800 Auto Hematology Analyzer (Mindray Biomedical Electronics Co. Ltd., Shenzhen, China) was used to perform routine blood tests. A SF-8100 Automated Coagulation Analyzer (Beijing Succeeder Technology Inc., Beijing, China) was used to detect the coagulation function. A BD FACSCanto II system (BD Biosciences, Franklin Lakes, NJ) was used to measure interleukins. A LABOSPECT008AS Automatic Analyzer (Hitachi HighTech, Tokyo, Japan) was used to analyze other blood biochemical indicators.

### Definition of variables.

Serum PAB levels were divided into the normal (PAB1) group and abnormal (PAB2) group based on the medical reference value of 150 to 400 mg/L. During this study, the PAB levels of all patients in the PAB2 group were less than 150 mg/L. A nutritional risk assessment was conducted within 48 hours of admission by two trained specialists using the NRS 2002 score,^25^^,^^26^ which considered the patient’s nutrition status (0 to 3 points), disease severity (2 to 3 points), and age (0 to 1 point). Participants 70 years or older scored 1 point, severely ill patients scored 2 points, and critically ill patients scored 3 points. Component scores were summed to yield a total score ranging from 2 to 7, with a higher score indicating a higher risk for malnutrition. Patients were considered at risk if their NRS score was more than 3 points.^27^ The prognosis for COVID-19 was categorized as death or discharge during this study. The criteria for discharge was in line with the *Diagnosis and Treatment of COVID-19* (trial version 8) created by the National Health Commission of China.^24^ The length of stay was the duration between admission and discharge (or death). Patients with symptoms of nausea, vomiting, or diarrhea were categorized into the gastrointestinal disorder group.

### Statistical analysis.

The Shapiro-Wilk test and Q-Q graph were used to test the normality of continuous variables. Normally distributed continuous data were described as the mean ± standard deviation (SD) or median (interquartile range values [IQR; 25th–75th percentile]). The means were compared using Student’s *t* test or the Mann-Whitney *U* test. Categorical variables were presented as frequency rates (%). The χ^2^ test or Fisher exact test were performed to compare the PAB1 and PAB2 groups. A multivariate linear regression was performed to explore the association of PAB with the NRS score (treated as a continuous variable), inflammatory factors, immune cells, and hospital length of stay. The corresponding correlation index (β) and its 95% confidence interval (CI) are presented. Logistic regression was used to assess the correlation of PAB with nutritional risk (binary variable) and the prognosis for COVID-19 patients using multivariate-adjusted odds ratios (ORs) with 95% CIs. A Kaplan-Meier survival analysis was conducted to determine if PAB is an independent predictor of mortality and to visualize the results. Moreover, a receiver-operating characteristic (ROC) curve was created to assess the prognostic power of the PAB level for mortality caused by COVID-19 pneumonia. A total of three models were implemented in the regression analysis. Model 1 was a crude unadjusted model; model 2 was adjusted for age, sex, and gastrointestinal disorder; and model 3 was adjusted for model 2 plus hypertension, diabetes, and cardiovascular disease. The confounding factors adjusted in the analysis were chosen based on the univariate analysis and previous studies.^2^^,^^19^^,^^28^^–^^31^ Mediation analyses with dichotomous outcomes were used to test whether there were some underlying factors that mediated the association between serum PAB and the prognosis.

All statistical analyses were performed using SPSS 26.0 (SPSS Inc., Chicago, IL) and GraphPad Prism 9.0. A mediation analysis was performed using PROCESS for SPSS with 5000 bootstrap resamples. Statistically significant differences were estimated using a two-tailed significance level of α = 0.05.

## RESULTS

### Basic characteristics and clinical features of COVID-19 patients.

A total of 408 patients were enrolled in this study. Of these patients, 183 had normal serum PAB levels (150–400 mg/L) and 225 had abnormal serum PAB levels (< 150 mg/L). Basic characteristics and clinical features of the participants are provided in Table 1. The average ages were 61.0 ± 12.6 years for all patients, 58.3 ± 12.4 years for the PAB1 group patients, and 63.2 ± 12.3 years for the PAB2 group patients. Of these patients, 210 (51.5%) were male, including 125 (55.6%) patients with reduced PAB levels. The three most common clinical symptoms were fever (336/408; 82.4%), cough (309/408; 75.7%), and reduced appetite (243/408; 59.6%); in addition, symptoms of fatigue (201/408; 49.3%) and dyspnea (168/408; 41.2%) were observed in nearly half of all COVID-19 patients. Furthermore, 156 (38.2%) COVID-19 patients had at least one underlying chronic disease, with hypertension and diabetes being the two most common. The average NRS score was 3.48 ± 1.01 points. The average hospital length of stay was 29.2 ± 11.3 days. Notably, 196 COVID-19 patients were at nutritional risk (NRS score ≥ 4) and 67.3% (132/196) of them had decreased PAB levels. Regarding disease severity, 316 (82.9%) patients were severely ill and 65 (17.1%) were critically ill. Compared with the PAB1 group, patients in the PAB2 group were significantly older (*P* < 0.001) and more likely to report fatigue (*P* = 0.011) and headache (*P* = 0.049). Furthermore, more patients in the PAB2 group were at nutritional risk (*P* < 0.001), were critically ill (*P* < 0.001), had longer hospital stays (*P* = 0.002), and were at higher risk for death (*P* < 0.001).

**Table 1:** Demographic characteristics and clinical features of participants with COVID-2019*

Variables	Total (*N* = 408)		PAB1 (*N* = 183)		PAB2 (*N* = 225)		*P* value†
Age, y (n)	61.0 ± 12.6	(408)	58.3 ± 12.4	(183)	63.2 ± 12.3	(225)	< 0.001
Sex, male, n (%)	210	(51.5)	85	(46.4)	125	(55.6)	0.067
Respiratory rate, bpm	20	(20–22)	20	(20–22)	20	(20–23)	0.129
Systolic pressure, mmHg	130	(120–142)	133	(12–145)	126	(119–140)	0.015
Diastolic pressure, mmHg	80	(72–89)	82	(73–90)	80	(72–86)	0.010
Signs and symptoms							
Fever, n (%)	336	(82.4)	147	(80.3)	189	(84.0)	0.659
Cough, n (%)	309	(75.7)	142	(77.6)	167	(74.2)	0.070
Sputum production, n (%)	144	(35.3)	64	(35.0)	80	(35.6)	0.908
Fatigue, n (%)	201	(49.3)	95	(51.9)	106	(47.1)	0.011
Myalgia, n (%)	105	(25.7)	49	(26.8)	56	(24.9)	0.529
Headache, n (%)	46	(11.3)	27	(14.8)	19	(8.4)	0.049
Dyspnea, n (%)	168	(41.2)	66	(36.1)	102	(45.3)	0.141
Gastrointestinal disorder, n (%)	106	(26.0)	47	(25.7)	59	(26.2)	0.902
Impaired appetite, n (%)	243	(59.6)	112	(61.2)	131	(58.2)	0.542
Any comorbidity							
Hypertension, n (%)	115	(28.2)	46	(25.1)	69	(30.7)	0.217
Diabetes, n (%)	47	(11.5)	16	(8.7)	31	(13.8)	0.113
Cardiovascular disease, n (%))	38	(9.3)	12	(6.6)	26	(11.6)	0.084
Pulmonary disease, n (%)	17	(4.2)	5	(2.7)	12	(5.3)	0.191
Cancer, n (%)	9	(2.2)	3	(1.6)	6	(2.7)	0.716
Chronic renal diseases, n (%)	10	(2.5)	7	(3.8)	3	(1.7)	0.195
History of surgery, n (%)	84	(20.6)	38	(20.8)	46	(20.4)	0.387
Drug allergy, n (%)	35	8.6	13	7.1	22	9.8	0.196
NRS score	3.48 ± 1.01	(372)	3.27 ± 0.86	(169)	3.66 ± 1.08	(203)	< 0.001
≤ 3, n (%)	212	52.0	119	56.1	93	43.9	< 0.001
≥ 4, n (%)	196	48.0	64	32.7	132	67.3	
The severity of disease							< 0.001
Severe, n (%)	316	82.9	154	91.1	162	76.4	
Critical, n (%)	65	17.1	15	8.9	50	23.6	
Outcomes							< 0.001
Discharge, n (%)	362	91.0	176	97.2	186	85.7	
Died, n (%)	36	9.0	5	2.6	31	14.3	
Hospital length of stay, days	29.2 ± 11.3	(398)	27.4 ± 10.6	(181)	30.8 ± 11.7	(217)	0.002

COVID-19 = coronavirus disease 2019; NRS = nutritional risk screening 2002; PAB = prealbumin; PAB1 = 150–400 mg/L; PAB2 = PAB < 150 mg/L.

* Continuous variables are presented as mean ± standard deviation (SD) or median (interquartile range) for normally and abnormally distributed continuous data; categorical variables are shown as n (%).

† *P* values are from the *t* test for normally distributed continuous data, from the Mann-Whitney *U* test for abnormally distributed continuous data, and from the χ^2^ test for categorical data.

**Figure 1.**
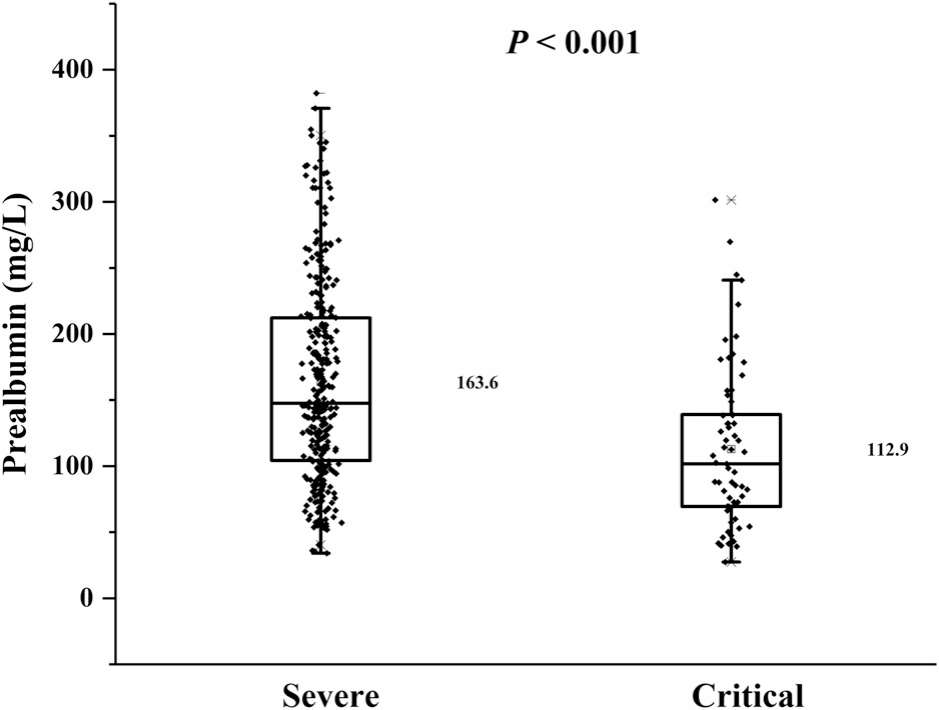
Distribution of prealbumin (PAB) among patients across severity of disease. The *P* value was calculated using Student’s *t* test.

As presented in Figure 1, the distribution of PAB was significantly different among patients stratified by disease severity. Critically ill patients have lower PAB levels (112.9 ± 61.0 mg/L, which is below the lower limit of the reference level) than severely ill patients (163.6 ± 75.9 mg/L). Therefore, the regression analysis during this study was conducted and presented based on different disease states.

### Laboratory characteristics of COVID-19 patients on admission.

Laboratory characteristics of participants are provided in Table 2. Patients with lower PAB levels exhibited a stronger inflammatory response with higher neutrophil counts, total B lymphocyte counts, C-reactive protein (CRP) levels, interleukin (IL)-6 levels, and IL-10 levels. Additionally, higher levels of globulin, glucose, and aspartate aminotransferase were also observed. However, patients in the PAB2 group had worse immunity status, with reduced lymphocyte counts, CD8^+^ T cell counts, and natural killer (NK) cell counts. Lower platelet counts, total protein levels, serum albumin levels, total cholesterol levels, triglyceride levels, high-density lipoprotein cholesterol levels, and low-density lipoprotein cholesterol levels were also observed in these patients.

**Table 2:** Laboratory characteristics on admission for participants with COVID-2019*

Variables	Total (*N* = 408)	PAB1 (*N* = 183)	PAB2 (*N* = 225)	*P* value†
Blood routine				
White blood cell count, 10^9^/L	5.76 (4.38–7.36)	5.63 (4.42–7.24)	5.92 (4.18–7.44)	0.814
Red blood cell count, 10^9^/L	4.10 (3.75–4.43)	4.15 (3.76–4.43)	4.07 (3.70–4.41)	0.216
Neutrophil count, 10^9^/L	4.01 (2.89–5.86)	3.74 (2.71–5.33)	4.37 (3.01–6.26)	0.014
Lymphocyte count, 10^9^/L	1.00 (0.72–1.36)	1.28 (0.92–1.62)	0.83 (0.58–1.08)	< 0.001
CD3 + T cell count, ×10^6^/L	73.5 (65.8–78.9)	75.1 (64.9–79.9)	72.3 (67.8–76.9)	0.140
CD4 + T cell count, ×10^6^/L	43. 7 (37.7–50.3)	42.7 (37.2–49.2)	44.0 (38.2–51.9)	0.331
CD8 + T cell count, ×10^6^/L	23.8 (17.1–29.7)	25.9 (20.2–30.3)	21.6 (15.1–27.2)	0.008
CD4 + T/CD8 + T	1.95 (1.42–2.76)	1.70 (1.24–2.33)	2.13 (1.51–3.38)	0.010
Total B lymphocyte count, 10^6^/L	11.3 (7.24–16.4)	9.78 (7.01–13.4)	14.1 (8.12–19.5)	0.007
NK cell count, 10^6^/L	7.44 (4.76–12.0)	8.49 (5.26–14.5)	7.00 (4.58–10.3)	0.134
Platelet count, 10^9^/L	225.0 (159.8–294.3)	244.0 (184.5–311.0)	197.0 (144.5–274.5)	< 0.001
Hemoglobin, g/L	126.0 (115.0–136.0)	128.0 (116.0–137.0)	125.0 (115.0–135.0)	0.291
Inflammatory markers				
Procalcitonin, ng/mL	0.07 (0.05–0.14)	0.06 (0.04–0.09)	0.10 (0.06–0.21)	< 0.001
C-reactive protein, mg/L	23.1 (4.69–63.7)	4.83 (1.41–16.2)	56.2 (26.9–89.0)	< 0.001
IL-2, pg/mL	2.37 (2.11–2.76)	2.31 (2.05–2.69)	2.41 (2.16–2.84)	0.224
IL-4, pg/mL	1.81 (1.41–2.48)	1.69 (1.35–2.32)	1.98 (1.45–2.69)	0.147
IL-6, pg/mL	5.64 (3.48–12.0)	4.49 (3.17–7.04)	7.37 (4.04–17.0)	0.014
IL-10, pg/mL	3.01 (2.42–3.83)	2.68 (2.27–3.70)	3.22 (2.61–4.31)	0.007
TNF-α, pg/mL	1.89 (1.57–2.54)	1.84 (1.57–2.35)	1.99 (1.55–2.59)	0.398
IFN-γ, pg/mL	1.76 (1.36–2.20)	1.69 (1.34–2.08)	1.88 (1.42–2.25)	0.245
Nutrition-related markers				
Total protein, g/L	62.4 (58.9–66.1)	63.0 (59.4–67.5)	62.1 (58.6–65.4)	0.015
Serum albumin level, g/L	62.4 (58.9 –66.1)	32.3 (29.5–35.6)	29.5 (26.0–32.7)	< 0.001
Globulin, g/L	31.1 (28.4–34.8)	30.2 (27.7–33.7)	31.7 (29.3–35.8)	< 0.001
Serum albumin:globulin	1.00 (0.80–1.20)	1.10 (0.90–1.20)	0.90 (0.80–1.10)	< 0.001
Serum urea nitrogen, mmol/L	4.67 (3.56–6.54)	4.78 (3.57–6.56)	4.58 (3.52–6.51)	0.792
Creatinine, μmol/L	68.8 (57.2–82.1)	68.8 (56.4–82.2)	69.1 (58.2–82.1)	0.888
Glucose, mmol/L	6.13 (5.35–7.91)	5.74 (5.18–7.06)	6.55 (5.65–8.71)	< 0.001
Total bilirubin, μmol/L	10.7 (8.03–14.3)	9.90 (7.30–12.7)	11.4 (8.60–15.9)	< 0.001
Total cholesterol, mmol/L	3.99 (3.40–4.57)	4.30 (3.71–5.00)	3.73 (3.24–4.39)	< 0.001
Triglyceride, mmol/L	1.33 (1.03–1.84)	1.63 (1.20–2.20)	1.16 (0.95–1.49)	< 0.001
HDL-C, mmol/L	0.89 (0.77–1.07)	0.91 (0.80–1.11)	0.86 (0.74–1.05)	0.021
LDL-C, mmol/L	2.35 (1.82–2.91)	2.47 (1.93–3.14)	2.23 (1.78–2.75)	0.019
AST, U/L	29.0 (21.0–43.0)	24.0 (18.0–36.0)	34.0 (25.0–49.0)	< 0.001
ALT, U/L	31.0 (20.0–51.0)	31.0 (19.0–52.0)	31.0 (20.0–49.5)	0.724

ALT = alanine aminotransferase; AST = aspartate aminotransferase; COVID-19 = coronavirus disease 2019; HDL-C = high-density lipoprotein cholesterol; IFN = interferon; IL = interleukin; IQR = interquartile range; LDL-C = low-density lipoprotein cholesterol; PAB = prealbumin; TNF = tumor necrosis factor.

PAB1 group: PAB + 150–400 mg/L; PAB2 group: PAB < 150 mg/L.

* Data are presented as median (interquartile range).

† *P* values are from Mann-Whitney *U* test for abnormally distributed continuous data.

### Associations among serum PAB, the prognosis, and hospital length of stay for COVID-19 patients according to different models.

The index for the goodness of fit of the model (Akaike information criterion [AIC]) was used to evaluate the model fit and to avoid overfitting. The ORs and AIC trends according to variations in model complexity are shown in Supplemental Figure 2. The AICs for models 1, 2, and 3 decreased constantly, which suggested that the fit of model 3 was the most stable. Table 3 displays the relationships among PAB, mortality, and hospital length of stay for COVID-19 patients. Compared with the PAB1 group, patients with PAB levels < 150 mg/L had a 248% higher likelihood of mortality (adjusted odds ratio [aOR], 3.48; 95% CI, 1.14–10.64) according to the adjusted model (model 3). Moreover, patients with lower PAB levels (< 150 mg/L) tended to have longer hospital stays according to the crude the model (β = 2.74; 95% CI, 0.45–5.03) and the multi-adjusted model (β = 3.20; 95% CI, 0.99–5.41). These results were confirmed by Kaplan-Meier survival estimates, which showed a higher likelihood for mortality with decreasing PAB concentrations (Supplemental Figure 3).

**Table 3:** Association of PAB with mortality and hospital length of stay for COVID-19 patients*

	OR (95% CI)		*P* value	β (95% CI)		*P* value
	Mortality			Hospital length of stay, days		
	PAB1	PAB2		PAB1	PAB2	
Model 1	1 (Ref)	5.87 (2.23–15.43)	< 0.001	0 (Ref)	2.74 (0.45–5.03)	0.019
Model 2	1 (Ref)	4.59 (1.71–12.29)	0.002	0 (Ref)	2.34 (0.01–4.68)	0.049
Model 3	1 (Ref)	3.48 (1.14–10.64)	0.029	0 (Ref)	3.20 (0.99–5.41)	0.005†

CI = confidence interval; COVID-19 = coronavirus disease 2019; OR = odds ratio; PAB = prealbumin.

Model 1: unadjusted.

Model 2: adjusted for age, sex, gastrointestinal disorder.

Model 3: adjusted for model 2 plus hypertension, diabetes, cardiovascular disease, and disease severity.

* Logistic regression models were used to analyze the association between serum PAB and the prognosis, and linear regression models were used to analyze the association between serum PAB and hospital length of stay. PAB was a binary variable classified as PAB1 (PAB = 150–400 mg/L) or PAB2 (PAB < 150 mg/L).

† Information of clinical outcome was added to model 3 additionally to evaluate the association between PAB and hospital length of stay.

**Figure 2.**
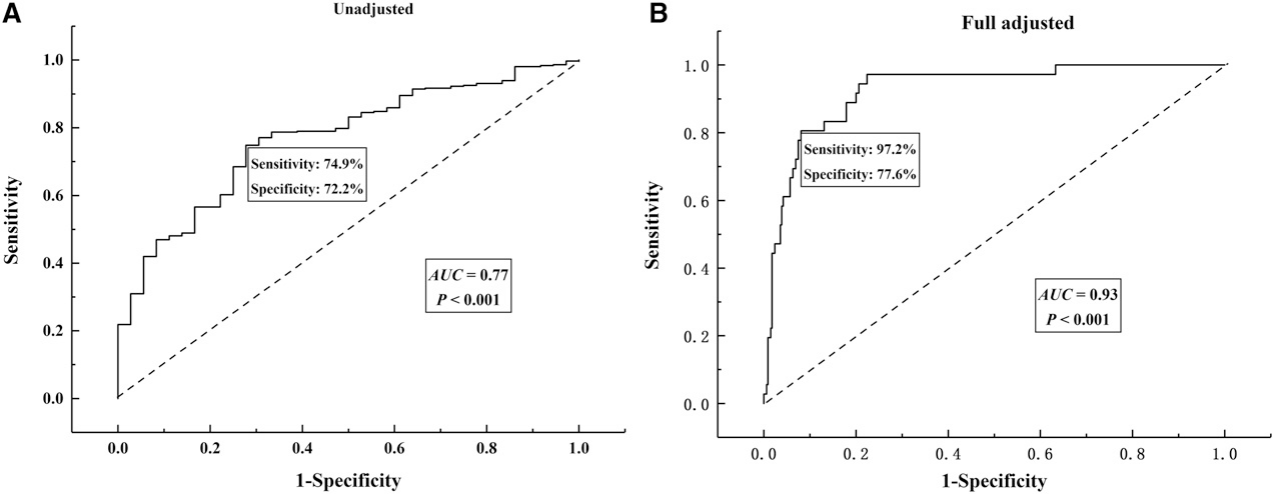
Receiver-operating characteristic (ROC) curve of prealbumin (PAB) for determining the prognosis of patients with coronavirus disease 2019 (COVID-19). (**A**) Unadjusted. (**B**) Adjusted for age, sex, gastrointestinal disorder, hypertension, diabetes, and cardiovascular disease.

The ROC for PAB used to determine the prognosis for COVID-19 patients is presented in Figure 2. Using PAB level as a sole predictor, the area under the receiver-operating characteristic (AUROC) curve was 0.77 (95% CI, 0.70–0.84; *P* < 0.001) (Figure 2A). After adjusting for potential confounders, the AUROC curve was 0.93 (95% CI, 0.89–0.97; *P* < 0.001), with sensitivity of 97.2% and specificity of 77.6% (Figure 2B), indicating that PAB has high diagnostic value for determining prognoses.

### Association between the serum PAB level and NRS score for COVID-19 patients.

The outcomes of the multiple linear regression analysis and logistic regression are presented in Supplemental Table 1. A negative association was observed between the PAB level and NRS score; this association was more pronounced for severely ill patients. According to the fully adjusted model (model 3), severely ill patients in the PAB2 group had a 2.18-fold higher nutritional risk compared with severely ill patients in the PAB1 group. Similar results were not observed for critically ill patients when using the crude model and the fully adjusted model.

### Association between serum PAB levels and inflammatory markers for COVID-19 patients.

Supplemental Table 2 shows the correlation between PAB levels and inflammatory markers stratified by disease severity. The PAB level was inversely correlated with C-reactive protein for COVID-19 patients. Compared with the PAB1 group, a 38.18-mg/L increase in the C-reactive protein concentration was observed in severely ill patients, and a 68.68-mg/L increase was observed in critically ill patients in the PAB2 group. A significant association was not observed between other inflammatory markers and PAB.

### Association between serum PAB levels and immune cell counts for COVID-19 patients.

Supplemental Table 3 presents the correlation between PAB levels and immune cell counts according to the severity of disease. For severe cases, decreased PAB levels were significantly associated with increased B lymphocyte counts (*P* < 0.05); however, they were not significant for critical cases. Moreover, patients with lower PAB concentrations have lower NK cell counts that are also more pronounced in severe cases. No significant association was observed between other immune cell counts and PAB.

### Mediation effect.

**Figure 3.**
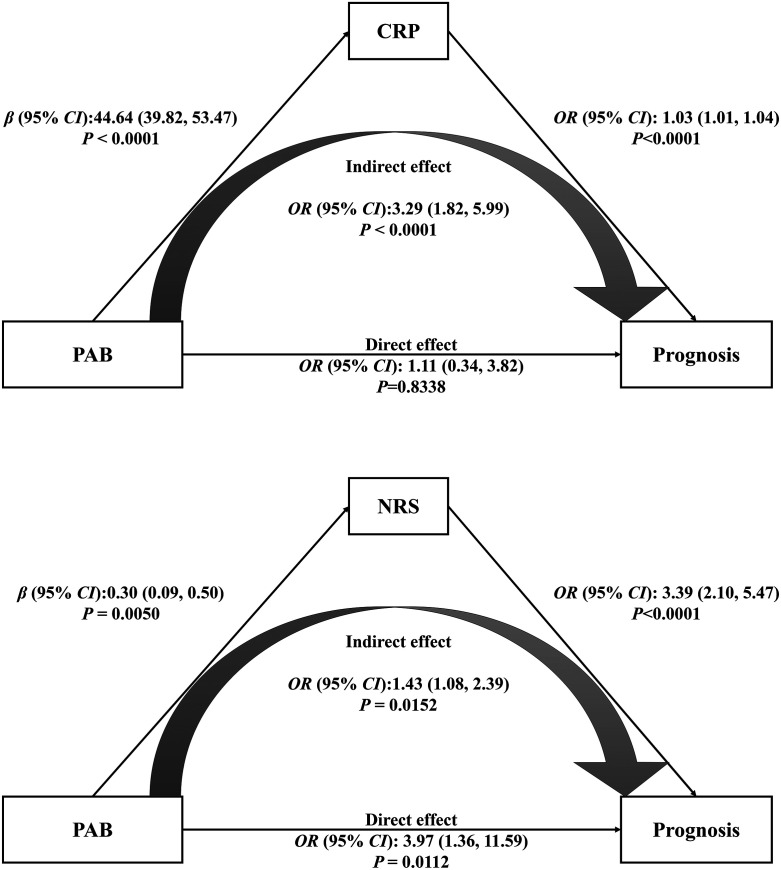
Mediation analysis of the relationship between prealbumin (PAB) and prognosis according to C-reactive protein (CRP) and nutrition risk screening 2002 (NRS) score. CI = confidence interval; OR = odds ratio. Adjusted for age, sex, gastrointestinal disorder, hypertension, diabetes, and cardiovascular disease.

To explore the roles of prognostic factors and their association with PAB, we conducted a mediation analysis. Based on the regression analysis, the NRS score, CRP levels, B lymphocyte counts, and NK cell counts were analyzed as mediators. Mediation effects were seen with the NRS score and CRP, but not with B lymphocyte and NK cell counts (data not shown). Figure 3 presents how CRP and the NRS mediated the relationship between PAB and the prognosis. The results of the regression analysis conducted to determine the mediation effect are depicted in Supplemental Table 4. With the mediation effect of CRP, the total effect of PAB on the prognosis was significant (total effect: aOR, 4.90; 95% CI, 1.80–13.20; *P* = 0.0019). The estimated aORs of the significant indirect effect mediated by CRP and the nonsignificant direct effect were 3.29 (95% CI, 1.82–5.99; *P* = 0.0017) and 1.11 (95% CI, 0.34–3.82; *P* = 0.8338). Similar mediation effects were also observed with the NRS score. The total effect, indirect effect, and direct effect mediated by the NRS score were significant. Corresponding aORs were 4.48 (95% CI, 1.68–12.30; *P* = 0.0029), 1.43 (1.08–2.39; *P* = 0.0152), and 3.97 (1.36–11.59; *P* = 0.0112).

**Figure 4.**
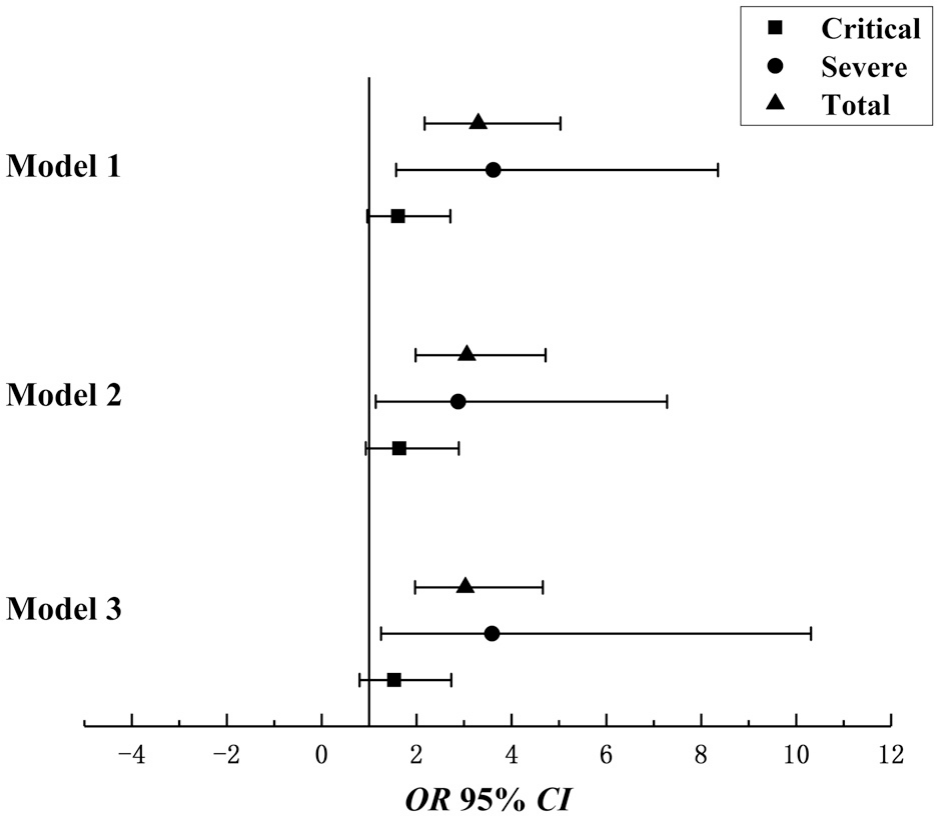
Multivariate-adjusted odds ratio (OR) and 95% confidence interval (CI) for C-reactive protein (CRP)/prealbumin (PAB) and prognosis. Model 1: unadjusted. Model 2: adjusted for age, sex, and gastrointestinal disorder. Model 3: adjusted for model 2 plus hypertension, diabetes, and cardiovascular disease.

Moreover, we analyzed the association of the CRP/PAB ratio with the prognosis for COVID-19 (Figure 4 and Supplemental Table 5). The estimated aORs were 3.03 (95% CI, 1.97–4.66) for all patients, 3.59 (95% CI, 1.25–10.31) for severely ill patients, and 1.53 (95% CI, 0.86–2.73) for critically ill patients. Supplemental Figure 4 shows the ROC curves of PAB and CRP/PAB for predicting the prognosis. PAB alone was a better indicator of the prognosis according to the AUROC curve, but CRP/PAB had higher specificity (77.6% versus 84.5%).

## DISCUSSION

The consistently increasing number of confirmed COVID-19 patients worldwide^5^^,^^32^ and considerable mortality of severely or critically ill patients^8^ alert clinicians to focus close attention on the clinical parameters of patients at the time of admission so an appropriate intervention can be performed during an early stage. In previous studies, the PAB level has been identified as a better indicator of the prognosis for COVID-19.^12^^,^^33^ A meta-analysis including 19 studies and 4616 COVID-19 patients suggested that severely ill patients and nonsurvivors had lower PAB levels, which were significantly associated with COVID-19 severity and mortality.^21^ Some studies have evaluated the power of serological biomarkers of inflammation for predicting disease severity.^34^ Neutrophil-to-lymphocyte ratios, CRP-to-prealbumin ratios (HsCPAR), and CRP-to-albumin ratios (HsCAR) were considered accurate biomarkers that could be used for predicting the prognosis.^34^ Few studies have been performed to explore the association of PAB with prognosis factors for COVID-19, however. As complementary research, this study provides a comprehensive analysis that aimed to explore the predictive power of PAB and its association with prognosis factors among severely ill and critically ill patients.

During this retrospective, observational study, the serum level of PAB was associated with the prognosis for COVID-19 patients and had good predictive power. Furthermore, obvious associations were observed among PAB and other prognosis factors such as nutritional risk, inflammatory markers, and immunity indicators. CRP and the NRS score had a mediation effect on the association of PAB and the prognosis. Our study findings suggest that the PAB concentration on admission is a biomarker for malnutrition and inflammation, which are crucially important for evaluating disease progression.

During this study, approximately 55.6% of patients had a lower PAB (< 150 mg/L) concentration, including 51.3% of severely ill patients and 76.9% of critically ill patients. The finding of a significant reduction in PAB for COVID-19 patients was consistent with the findings of other studies.^10^^,^^12^^,^^35^ Our study suggests that the PAB level of COVID-19 patients could act as a surrogate biomarker for disease progression and a good predictor of the prognosis.^10^^,^^12^ A study that evaluated the change in PAB from admission to discharge for patients who did and did not recover from COVID-19 proved that the PAB level is a better predictor than hypersensitive CRP levels, procalcitonin levels, d-dimer levels, and lymphocyte counts when monitoring disease progression during viral infection.^12^ However, the negative associations of the PAB level with mortality and hospital stay disappeared when stratified by disease severity on admission. There are underlying reasons for this. First, the reduction in PAB was significantly associated with the severity of COVID-19, which is a critical factor for the prognosis.^11^^,^^36^ Therefore, the disease severity might interfere with the relationship between PAB and the prognosis. Second, bias was inevitable because of the limited number of critically ill patients involved. A previous study involving patients managed in the ICU with total parenteral nutrition demonstrated that an increase in the PAB level did not indicate a better prognosis for critically ill patients.^37^ Although the conclusion of that study^37^ was in accordance with ours, that study was limited by its sample size (a total of 44 patients). Another study considered PAB as a prognostic indicator for critically ill patients in the ICU.^13^ To further explore the predictive power of PAB for critically ill COVID-19 patients, well-designed trials involving a large population are needed.

Malnutrition, immunity status, and inflammatory response were the main prognostic factors for COVID-19.^7^^,^^18^^,^^36^ Therefore, we further evaluated the associations among PAB and these prognostic factors during this study. A tremendous nutritional risk was noticed for severely ill and critically ill patients with lower PAB concentrations. A meta-regression performed among 52,911 participants in 2017 indicated that individuals with lower PAB levels were at higher risk for malnutrition.^38^ PAB has been selected as a reliable marker for evaluating malnutrition^39^ and the effects of aggressive nutritional support^23^^,^^39^ for severely ill and critically ill patients. Moreover, the NRS score was associated with the prognosis by partially mediating the path between PAB and the prognosis for COVID-19 patients. However, during our study, PAB was not significantly associated with the nutritional risk for critically ill patients. A previous study involving 80 critically ill patients in the ICU also found that PAB was not suitable as the sole nutrition indicator when evaluating the nutritional status of critically ill patients.^23^

The “cytokine storm” phenomenon, which is related to viral pneumonia, has been suggested to occur with COVID-19,^6^^,^^40^^,^^41^ thereby increasing the severity of disease. Regarding SARS, the viral load did not lead to worsening symptoms; however, an overexuberant inflammatory response was common.^42^ Previous studies have reported abnormal levels of IL-2, IL-6, IL-7, IL-10, CRP, and tumor necrosis factor-α in COVID-19 patients.^7^^,^^19^^,^^28^ In fact, the expression of CRP is usually low in healthy people and increases to a higher level during acute inflammatory responses.^43^ Liu et al.^36^ suggested that CRP could effectively predict outcomes of COVID-19 patients. During our study, increased CRP levels in COVID-19 patients emerged with reduced PAB concentrations. Our further study of the mediation effect of CRP indicated that the PAB level could influence the prognosis for COVID-19 patients via CRP-mediated inflammation. The positive association between CRP and PAB combined and mortality verified our speculation.

Immune responses are important to the progression of COVID-19. The immune response will be triggered by host factors when viral infection occurs.^44^^,^^45^ During this study, patients with PAB levels <150 mg/L had higher B cell counts but lower NK cell counts, especially severely ill patients. Anti-S antibodies had an important role in inhibiting viral entrance in permissive cells but potentiated the infection by binding to IgG Fc receptor II on B cells.^46^ Previous exposure to other coronaviruses contributed to antibody-dependent enhancement.^47^ Antibody-dependent enhancement is associated with the severity of coronavirus infections,^47^ which may be a possible explanation for the relatively higher level of B cells in the PAB2 group. Similar to T cells, NK cell reduction is common in COVID-19 patients.^48^ The increased neutrophil counts in the PAB2 group reflected an inadequate host response to coronavirus aggression,^49^ in line with the aforementioned changes in immune cells. The mechanism causing the differences in the associations between PAB and immune cells in severely ill and critical ill cases requires further study.

Our study was the first to explore the associations of PAB, the prognosis, and risk factors for severely ill and critically ill COVID-19 cases. Furthermore, we explored the potential mechanism for PAB and the prognosis. We also observed mediation effects of CRP and the NRS score. The findings of this study highlight the important role of PAB in monitoring disease progression.

This study had some limitations. First, the design of this observational study limited the ability to predict any cause-and-effect relationship. Therefore, a cohort study involving dynamic monitoring of PAB levels is required to further investigate the role of PAB in the progression of COVID-19. Next, during data collection, critically ill patients admitted to the ICU were not enrolled in the analysis, thus leading to the selection bias. Finally, because of the absence of laboratory data for some patients, the absolute correlation between PAB and other biochemistry markers could have been missed. A larger, population-based, multicenter cohort will provide more conclusive and integral data than single-center studies.

## CONCLUSION

In summary, the PAB level is a convenient and valid tool for assessing the prognosis of COVID-19 patients. CRP and the NRS score mediated the association between the PAB level and the prognosis. For COVID-19 patients, nutritional support and inflammation detection may be a necessary component of their treatment plan.

## Supplemental tables and figures


Supplemental materials

## Notes

† These authors contributed equally to this work.
