# Supplementary material for: Role of Prealbumin in Predicting the Prognosis of Severely and Critically Ill COVID-19 Patients
Source: Am J Trop Med Hyg. 2021 Aug 9;105(3):718–26. doi: 10.4269/ajtmh.21-0234 (PMC8592333; doi:10.4269/ajtmh.21-0234)
Supplement: Supplementary file 1 [file tpmd210234.SD1.docx]

| **Supplemental Table S1.** **Association between serum PAB and NRS score of severely and critically ill patients with COVID-2019^a^.** | | | | | | | | |
| --- | --- | --- | --- | --- | --- | --- | --- | --- |
| **Variables** | ***β*(95% *CI*)** | | | ***P*-value** | ***aOR* (95% *CI*)** | | | ***P*-value** |
|  | **NRS score (continuous variable)** | | |  | **NRS score (binary variable)** | | |  |
|  | **Total** | **PAB1** | **PAB2** |  | **Total** | **PAB1** | **PAB2** |  |
| Model 1 |  |  |  |  |  |  |  |  |
| Total | 381 | 0 (Ref) | 0.39 (0.19, 0.60) | **<0.001** | 381 | 1 (Ref) | 2.64 (1.76, 3.95) | **< 0.001** |
| Severe cases | 316 | 0 (Ref) | 0.29 (0.12, 0.47) | **0.001** | 316 | 1 (Ref) | 2.26 (1.43, 3.58) | **< 0.001** |
| Critical cases | 65 | 0 (Ref) | -0.09 (-1.03, 0.84) | 0.842 | 65 | 1 (Ref) | 1.54 (0.34, 6.86) | 0.574 |
| Model 2 |  |  |  |  |  |  |  |  |
| Total | 381 | 0 (Ref) | 0.27 (0.06, 0.47) | **0.011** | 381 | 1 (Ref) | 2.43 (1.61, 3.68) | **< 0.001** |
| Severe cases | 316 | 0 (Ref) | 0.17 (0.00, 0.34) | **0.050** | 316 | 1 (Ref) | 2.13 (1.33, 3.41) | **0.002** |
| Critical cases | 65 | 0 (Ref) | -0.126 (-1.07, 0.82) | 0.791 | 65 | 1 (Ref) | 1.40 (0.30, 6.55) | 0.669 |
| Model 3 |  |  |  |  |  |  |  |  |
| Total | 381 | 0 (Ref) | 0.27 (0.06, 0.47) | **0.010** | 381 | 1 (Ref) | 2.46 (1.62, 3.72) | **< 0.001** |
| Severe cases | 316 | 0 (Ref) | 0.17 (0.01, 0.35) | **0.048** | 316 | 1 (Ref) | 2.18 (1.35, 3.52) | **0.001** |
| Critical cases | 65 | 0 (Ref) | -0.09 (-1.00, 0.81) | 0.837 | 65 | 1 (Ref) | 2.74 (0.41, 18.36) | 0.301 |

*CI* confidence interval; COVID-19, coronavirus disease 2019; NRS, Nutritional Risk Screening 2002; *OR*, odds ratio; PAB, prealbumin; PAB1, 150 mg/L≤ PAB≤400 mg/L; PAB2, PAB < 150 mg/L.

Model 1: un-adjusted;

Model 2: adjusted for age, gender, gastrointestinal disorder;

Model 3: adjusted for Model2 + hypertension, diabetes and cardiovascular diseases.

^a^ Logistic regression models were used to analyze the association between serum PAB and NRS (NRS ≤ 3 or NRS ≥ 4), and linear regression models were used to analyze the association between serum PAB and NRS score.

| **Supplemental Table S2.*β*and 95% *CI* for inflammatory factors according to serum PAB concentration of severely and critically ill patients with COVID-2019.** | | | | | | | | | | | |
| --- | --- | --- | --- | --- | --- | --- | --- | --- | --- | --- | --- |
| **Variables** | ***β*(95% *CI*)** | | | | | | | | | | |
|  | **Model1** | | |  | **Model2** | | |  | **Model3** | | |
|  | Total | Severe | Critical |  | Total | Severe | Critical |  | Total | Severe | Critical |
| IL-2,pg/mL | 0.03 (-0.15, 0.21) | -0.01 (-0.21, 0.18) | -0.01 (-1.16, 1.14) |  | -0.02 (-0.21, 0.16) | -0.07 (-0.26, 0.13) | -0.07 (-1.50, 1.36) |  | -0.04 (-0.23, 0.15) | -0.11 (-0.31, 0.08) | -0.19 (-1.58, 1.20) |
| *P*_-value_ | 0.739 | 0.889 | 0.985 |  | 0.818 | 0.480 | 0.911 |  | 0.672 | 0.237 | 0.756 |
| IL-4,pg/mL | -0.06 (-0.63, 0.51) | -0.13 (-0.79, 0.52) | 0.009 (-2.00, 2.17) |  | -0.17 (-0.75, 0.41) | -0.27 (-0.93, 0.40) | 0.15 (-2.43, 2.74) |  | -0.14 (-0.74, 0.45) | -0.24 (-0.93, 0.45) | -0.01 (-2.96, 2.94) |
| *P*_-value_ | 0.835 | 0.684 | 0.930 |  | 0.557 | 0.427 | 0.897 |  | 0.638 | 0.483 | 0.993 |
| IL-6,pg/mL | 23.01 (-3.69, 49.71) | 8.42 (-5.21, 22.04) | 68.13 (-283.17, 419.44) |  | 21.24 (-6.80, 49.27) | 10.65 (-3.53, 24.82) | 25.88 (-397.64, 449.40) |  | 26.08 (-0.99, 53.16) | 14.74 (0.83, 28.64) | -35.29 (-370.67, 300.09) |
| *P*_-value_ | 0.090 | 0.223 | 0.682 |  | 0.339 | 0.139 | 0.894 |  | 0.059 | 0.038 | 0.811 |
| IL-10,pg/mL | 0.82 (-0.10, 1.74) | 0.23 (-0.28, 0.74) | 1.80 (-9.76, 13.36) |  | 0.79 (-0.19, 1.77) | 0.03 (-0.47, 0.53) | -0.21 (-12.33, 11.91) |  | 0.81 (-0.20, 1.81) | -0.01 (-0.52, 0.51) | -0.58 (-14.79, 13.64) |
| *P*_-value_ | 0.081 | 0.374 | 0.742 |  | 0.112 | 0.908 | 0.970 |  | 0.113 | 0.991 | 0.926 |
| TNF-α,pg/mL | -0.05 (-0.35, 0.26) | -0.10 (-0.44, 0.24) | -0.11 (-1.63, 1.41) |  | -0.12 (-0.43, 0.20) | -0.16 (-0.52, 0.19) | -0.08 (-1.91, 1.75) |  | -0.15 (-0.47, 0.18) | -0.23 (-0.60, 0.13) | -0.23 (-2.22, 1.77) |
| *P*_-value_ | 0.752 | 0.558 | 0.878 |  | 0.474 | 0.363 | 0.924 |  | **0.001** | 0.199 | 0.797 |
| INF-γ,pg/mL | -0.11 (-0.58, 0.35) | -0.16 (-0.70, 0.37) | -0.09 (-1.64, 1.47) |  | -0.18 (-0.05, 2.93) | -0.22 (-0.78, 0.34) | -0.17 (-2.04, 1.70) |  | -0.21 (-0.71, 0.28) | -0.30 (-0.88, 0.29) | -0.34 (-2.24, 1.56) |
| *P*_-value_ | 0.632 | 0.549 | 0.908 |  | 0.058 | 0.431 | 0.840 |  | 0.396 | 0.314 | 0.686 |
| Procalcitonin, ng/mL | 0.02 (-0.11, 0.16) | -0.01 (-0.14, 0.12) | -0.15 (-0.60, 0.30) |  | 0.01 (-0.13, 0.14) | -0.02 (-0.15, 0.12) | -0.16 (-0.62, 0.30) |  | 0.01 (-0.13, 0.15) | -0.02 (-0.16, 0.12) | -0.16 (-0.63, 0.31) |
| *P*_-value_ | 0.754 | 0.871 | 0.500 |  | 0.950 | 0.797 | 0.489 |  | 0.909 | 0.816 | 0.490 |
| C-reactive protein, mg/L | 49.18 (42.39, 55.97) | 40.74 (34.54, 46.94) | 65.86 (41.66, 90.05) |  | 46.37 (39.62, 53.12) | 38.45 (32.34, 44.57) | 66.02 (41.33, 90.70) |  | 46.67 (39.87, 53.48) | 38.18 (32.00, 44.36) | 68.68 (44.45, 92.92) |
| *P*_-value_ | **< 0.001** | **< 0.001** | **< 0.001** |  | **< 0.001** | **< 0.001** | **< 0.001** |  | **< 0.001** | **< 0.001** | **< 0.001** |

*β* coefficient of regression, *CI* confidence interval COVID-19, coronavirus disease 2019; PAB, prealbumin, PAB was a binary variable classified into PAB1 (150 mg/L≤ PAB≤400 mg/L) and PAB2 (PAB < 150 mg/L)..

Model 1: un-adjusted;

Model 2: adjusted for age, gender, gastrointestinal disorder;

Model 3: adjusted for Model2 + hypertension, diabetes and cardiovascular diseases.

| **Supplemental Table S3.*β*and 95% *CI* for immune cells according to serum PAB concentration of severely and critically ill patients with COVID-2019.** | | | | | | | | | | | |
| --- | --- | --- | --- | --- | --- | --- | --- | --- | --- | --- | --- |
| **Variables** | ***β*(95% *CI*)** | | | | | | | | | | |
|  | **Model** | | |  | **Model2** | | |  | **Model3** | | |
|  | Total | Severe | Critical |  | Total | Severe | Critical |  | Total | Severe | Critical |
| CD3^+^T cells count, × 10^6^/L | -2.03 (-5.34, 1.28) | -1.74 (-5.01, 1.54) | -12.22 (-41.01, 16.57) |  | -0.94 (-4.26, 2.38) | -0.79 (-4.01, 2.49) | -9.93 (-41.44, 21.58) |  | -0.62 (-4.00, 2.76) | -0.45 (-3.85, 2.95) | -9.94 (-39.00, 19.11) |
| *P*_-value_ | 0.226 | 0.296 | 0.382 |  | 0.576 | 0.636 | 0.508 |  | 0.716 | 0.792 | 0.463 |
| CD4^+^T cells count, × 10^6^/L | 1.88 (-1.43, 5.19) | 1.52 (-1.97, 5.01) | -5.35 (-29.38, 18.69) |  | 1.99 (-1.45, 5.43) | 1.56 (-2.01, 5.13) | -2.57 (-29.55, 24.40) |  | 2.47 (-1.05, 5.98) | 2.26 (-1.40, 5.92) | -2.31 (-32.62, 28.00) |
| *P*_-value_ | 0.263 | 0.389 | 0.644 |  | 0.254 | 0.388 | 0.840 |  | 0.167 | 0.223 | 0.868 |
| CD8^+^T cells count, × 10^6^/L | -3.21 (-6.36, -0.06) | -2.32 (-5.62, 0.99) | -6.19 (-29.37, 16.99) |  | -2.64 (-5.76, 0.40) | -1.77 (-4.90, 1.37) | -6.96 (-31.25, 17.33) |  | -2.79 (-5.86, 0.27) | -2.07 (-5.32, 1.18) | -7.09 (-32.42, 18.24) |
| *P*_-value_ | **0.046** | 0.167 | 0.579 |  | 0.088 | 0.267 | 0.546 |  | 0.074 | 0.209 | 0.547 |
| CD4^+^T/CD8+T | 0.74 (0.18, 1.31) | 0.45 (0.04, 0.86) | 1.40 (-5.44, 8.23) |  | 0.61 (0.04, 1.18) | 0.38 (-0.02, 0.77) | 2.01 (-5.15, 9.17) |  | 0.63 (0.05, 1.21) | 0.42 (0.01, 0.83) | 2.07 (-6.29, 10.42) |
| *P*_-value_ | **0.011** | **0.032** | 0.671 |  | **0.036** | 0.064 | 0.554 |  | **0.033** | **0.047** | 0.593 |
| Total B lymphocytes count, 10^6^/L | 4.60 (1.67, 7.54) | 3.84 (1.12, 6.56) | 2.79 (-25.93, 31.52) |  | 4.54 (1.54, 7.53) | 3.65 (0.92, 6.38) | -3.18 (-33.91, 27.55) |  | 4.28 (1.30, 7.26) | 3.27 (0.48, 6.07) | -4.02 (-30.65,22.61) |
| *P*_-value_ | **0.002** | **0.006** | 0.839 |  | **0.003** | **0.009** | 0.827 |  | **0.005** | **0.022** | 0.743 |
| NK cells count, 10^6^/L | -2.35 (-4.46, -0.24) | -2.36 (-4.65, -0.07) | 6.41 (-6.76, 19.58) |  | -2.95 (-5.05, -0.84) | -2.78 (-5.04, -0.51) | 9.82 (-2.63, 22.28) |  | -3.06 (-5.21, -0.19) | -2.86 (-5.50, -1.04) | 10.41 (-3.51, 24.33) |
| *P*_-value_ | **0.030** | **0.044** | 0.318 |  | **0.006** | **0.017** | 0.112 |  | **0.006** | **0.005** | 0.127 |

*β* coefficient of regression, *CI* confidence interval; COVID-19, coronavirus disease 2019; PAB, prealbumin, PAB was a binary variable classified into PAB1 (150 mg/L≤ PAB≤400 mg/L) and PAB2 (PAB < 150 mg/L)..

Model 1: un-adjusted;

Model 2: adjusted for age, gender, gastrointestinal disorder;

Model 3: adjusted for Model2 + hypertension, diabetes and cardiovascular diseases.

| **Supplemental Table S4.** **Mediation analysis of the relationship between PAB and prognosis by CRP^a^.** | | | | | | |
| --- | --- | --- | --- | --- | --- | --- |
| **Parameters** | **CRP** | | | **NRS** | | |
|  | ***β*(95% *CI*)** | ***aOR* (95% *CI*)** | ***P-value*** | ***β(95% CI)*** | ***aOR* (95% *CI*)** | ***P-value*** |
| Total effect | 1.59 (0.59, 2.58) | 4.90 (1.80, 13.20) | 0.0017 | 1.51 (0.52, 2.51) | 4.48 (1.68, 12.30) | 0.0029 |
| Indirect effect path ab | 1.19 (0.60, 1.79) | 3.29 (1.82, 5.99) | < 0.0001 | 0.36 (0.08, 0.87) | 1.43 (1.08, 2.39) | 0.0152 |
| Path a | 46.64 (39.82, 53.47) | -- | < 0.0001 | 0.30 (0.09, 0.50) | -- | 0.0050 |
| Path b | 0.03 (0.01, 0.04) | 1.03 (1.01, 1.04) | < 0.0001 | 1.22 (0.74, 1.70) | 3.39 (2.10, 5.47) | < 0.0001 |
| Direct effect path c’ | 0.13 (-1.08, 1.34) | 1.11 (0.34, 3.82) | 0.8338 | 1.38 (0.31, 2.45) | 3.97 (1.36, 11.59) | 0.0112 |
| PE^b^ | 74.84%^c^ | | | 23.84% ^d^ | | |

CRP, C-reactive protein; NRS, nutrition risk score; PAB, prealbumin.

^a^ Adjusted for age, gender, gastrointestinal disorder, hypertension, diabetes and cardiovascular diseases. In the analysis, PAB was a binary variable classified into PAB1 (150 mg/L≤ PAB≤400 mg/L) and PAB2 (PAB < 150 mg/L).

^b^ PE = indirect effect/total effect.

^c^ Fully mediated.

^d^ Partially mediated.

Path a indicates the path from the PAB level to CRP or NRS level (Mediator);

Path b indicates the path from CRP or NRS to prognosis;

Path ab indicates the indirect effect of PAB prognosis mediated by CRP or NRS;

Path c’ indicates the direct effect of PAB on prognosis.

| **Supplemental Table S5. a*OR* and 95% *CI* between CRP/PAB and prognosis.** | | | | | | |
| --- | --- | --- | --- | --- | --- | --- |
| **CRP/PAB** | **Model 1** | | **Model 2** | | **Model 3** | |
|  | ***aOR* (95% *CI*)** | ***P-*value** | ***aOR* (95% *CI*)** | ***P-*value** | ***aOR* (95% *CI*)** | ***P-*value** |
| Total | 3.30 (2.17, 5.03) | **< 0.001** | 3.06 (1.98, 4.72) | **< 0.001** | 3.03 (1.97, 4.66) | **< 0.001** |
| Severe | 3.62 (1.57, 8.35) | **0.003** | 2.88 (1.14, 7.28) | **0.026** | 3.59 (1.25, 10.31) | **0.017** |
| Critical | 1.61 (0.96, 2.71) | 0.073 | 1.64 (0.93, 2.89) | 0.088 | 1.53 (0.86, 2.73) | 0.149 |

*CI*, confidence ratio; CRP, C-reactive protein; CRP/PAB, the ratio of CRP and PAB; *OR*, odds ratio; PAB, prealbumin.

Model 1: un-adjusted;

Model 2: adjusted for age, gender, gastrointestinal disorder;

Model 3: adjusted for Model2 + hypertension, diabetes and cardiovascular diseases.


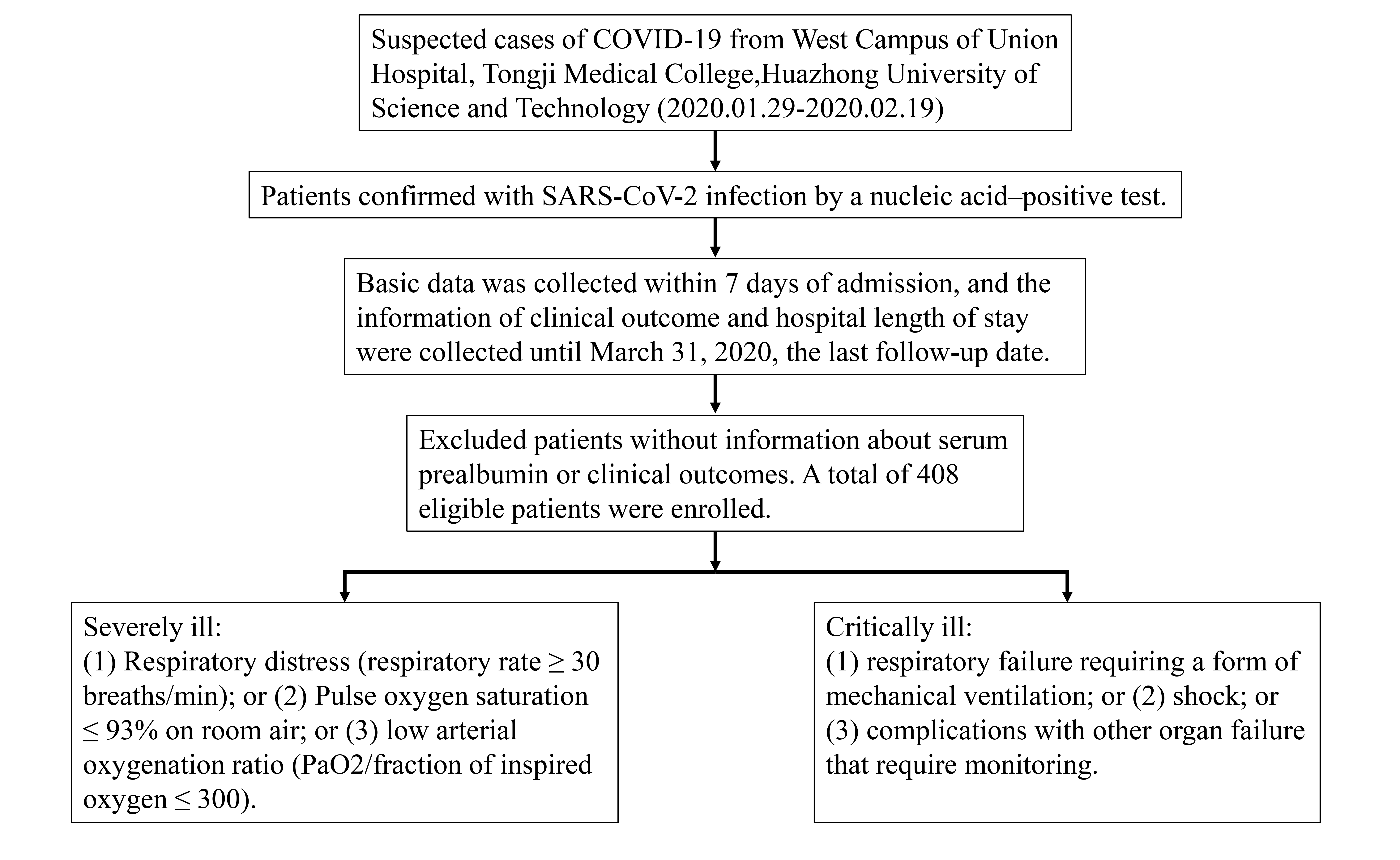


**Supplemental Figure 1. The flow chart to enroll patients eligible.**

**

**

**Supplemental Figure 2. Trend of OR and AIC according to the variation of model complexity.**

AIC, index of goodness fit of model; *CI*, confidence interval; *OR*, odds ratio.

Model 1: un-adjusted;

Model 2: adjusted for age, gender, gastrointestinal disorder;

Model 3: adjusted for Model2 + hypertension, diabetes, cardiovascular diseases and disease severity.


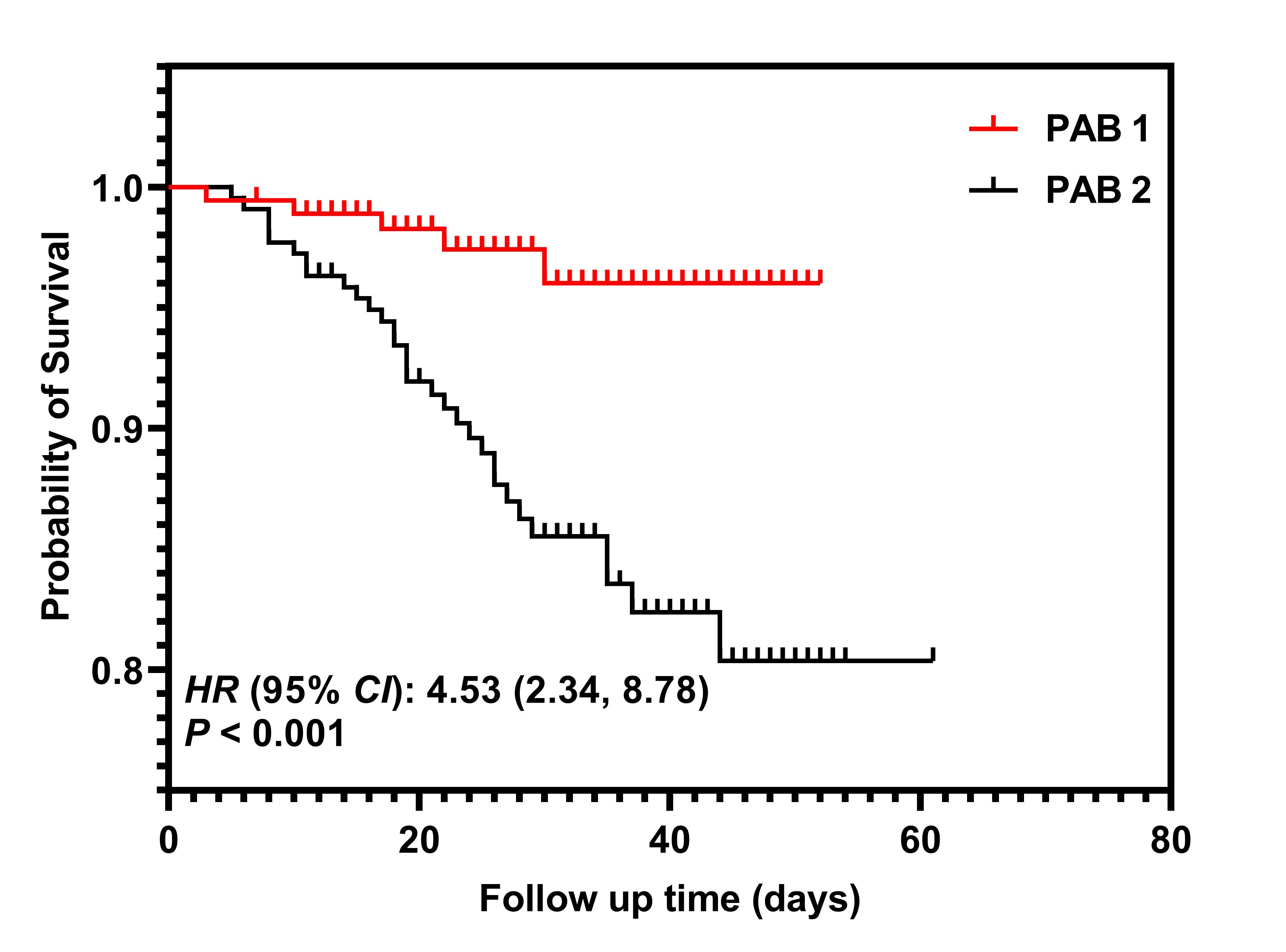


**Supplemental Figure 3. Survival of severely and critically ill patients with COVID-19, stratified by the PAB within the follow up period (*P* < 0.001).**

*CI*, confidence ratio; COVID-19, coronavirus disease 2019; *HR*, hazards ratio; PAB, prealbumin..

In the analysis, PAB was a binary variable classified into PAB1 (150 mg/L≤ PAB≤400 mg/L) and PAB2 (PAB < 150 mg/L).


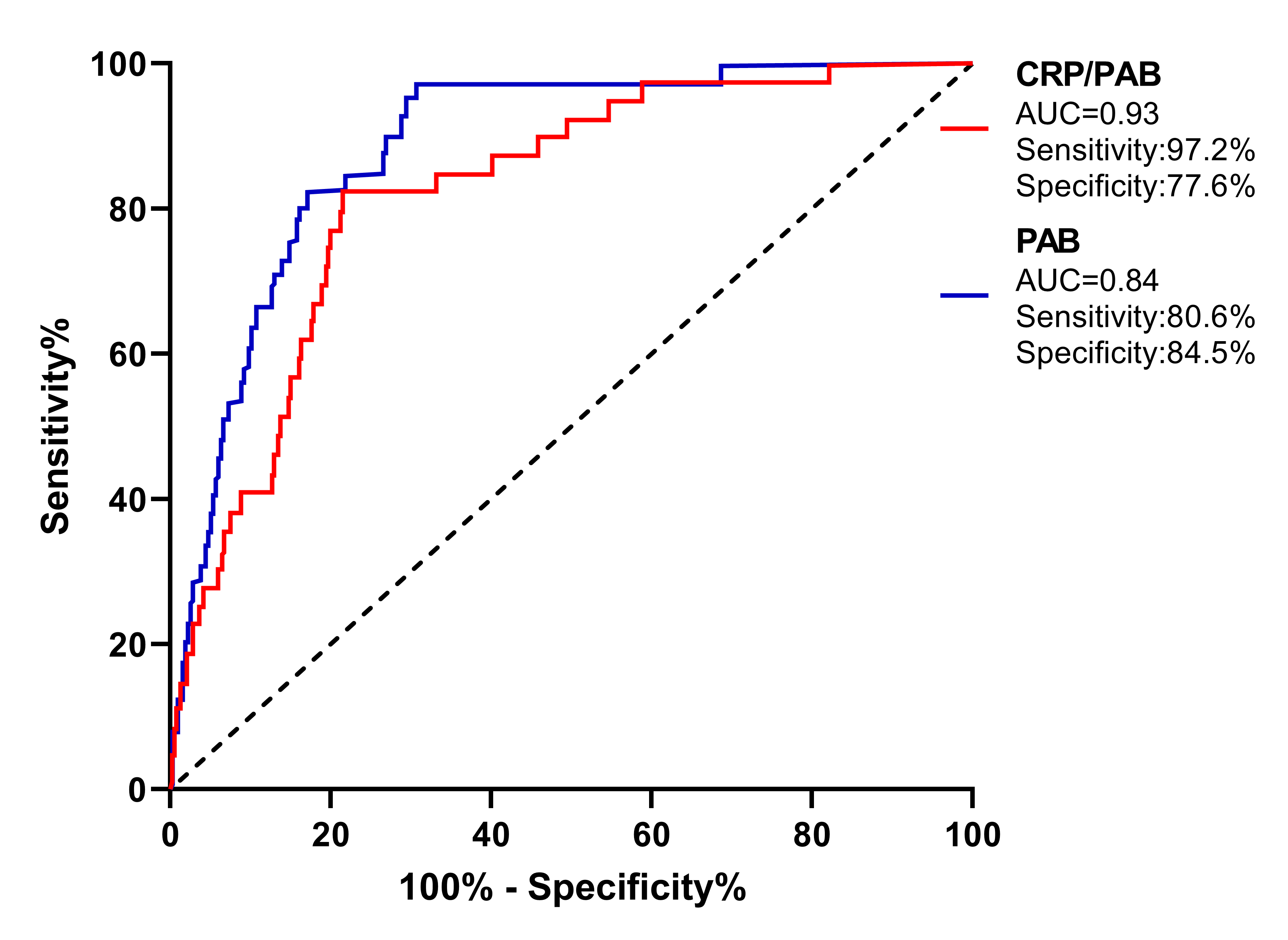


**Supplemental Figure 4. ROC curves of PAB and CRP/PAB for the prognosis of COVID-19 patients ^a^.**

AUC, area under the curve; COVID-19, coronavirus disease 2019; CRP, C-reactive protein; CRP/PAB, the ratio of CRP and PAB; PAB, prealbumin.

^a^ Adjusted for age, gender, gastrointestinal disorder hypertension, diabetes and cardiovascular diseases.
